# *PEMer*: a computational framework with simulation-based error models for inferring genomic structural variants from massive paired-end sequencing data

**DOI:** 10.1186/gb-2009-10-2-r23

**Published:** 2009-02-23

**Authors:** Jan O Korbel, Alexej Abyzov, Xinmeng Jasmine Mu, Nicholas Carriero, Philip Cayting, Zhengdong Zhang, Michael Snyder, Mark B Gerstein

**Affiliations:** 1 Gene Expression Unit, European Molecular Biology Laboratory (EMBL), Meyerhofstr., Heidelberg, 69117, Germany; 2 EMBL Outstation Hinxton, EMBL-European Bioinformatics Institute (EMBL-EBI), Wellcome Trust Genome Campus, Hinxton, Cambridge, CB10 1SA, UK; 3 Molecular Biophysics and Biochemistry Department, Yale University, Whitney Ave, New Haven, CT 06520, USA; 4 Department of Molecular, Cellular, and Developmental Biology, Yale University, Whitney Ave, New Haven, CT 06520, USA; 5 Department of Computer Science, Yale University, Prospect Street, New Haven, CT 06511, USA; 6 Program in Computational Biology and Bioinformatics, Yale University, Whitney Ave, New Haven, CT 06520, USA

## Abstract

Personal-genomics endeavors, such as the 1000 Genomes project, are generating maps of genomic structural variants by analyzing ends of massively sequenced genome fragments. To process these we developed *Paired-End Mapper *(*PEMer*; ). This comprises an analysis pipeline, compatible with several next-generation sequencing platforms; simulation-based error models, yielding confidence-values for each structural variant; and a back-end database. The simulations demonstrated high structural variant reconstruction efficiency for *PEMer*'s coverage-adjusted multi-cutoff scoring-strategy and showed its relative insensitivity to base-calling errors.

## Abstract 2

Paired-End Mapper (PEMer) enables mapping of genomic structural variants at considerably enhanced sensitivity, specificity and resolution over previous approaches.

## Rationale

Following the sequencing of the genomes of hundreds of species over the past years, measuring variation within individuals of a species - such as across human beings - has become a center of attention in genomics [1]. While it was long assumed that most of the variation in our genomes is due to single nucleotide polymorphisms (SNPs), the relative importance of another form of genomic variation has been recognized more recently: these are structural variants (SVs), frequently referred to as copy-number variants (CNVs), and here defined as kilobase to megabase sized deletions, insertions, duplications, and inversions. SVs presumably contribute to more base-pair differences between individuals than SNPs [2,3]. Furthermore, they may have considerable effects on human phenotypic variation [2] by causing common 'normal' phenotypic differences [4,5] and contributing to disease susceptibility [6-9].

A necessary prerequisite for identifying the functional impact of SVs on the genome is the construction of a comprehensive, high-resolution map of SVs across many individuals. However, relatively few approaches are available so far that allow mapping SVs at high resolution and in a cost-efficient manner. Previously, computational approaches have been described that enable detection of SVs at high resolution by either evaluating SNP genotyping information (for example, [10,11]), scoring high-density microarray platforms [12-15], measuring DNA sequence read densities [16], detecting split sequence reads [17], or comparing different human genome assemblies [18-20]. Each of these complementary approaches enables the identification of at least a subset of SVs at a reasonable confidence level. However, each method also has drawbacks in terms of overall sensitivity, effective resolution, or efficiency.

Recent surveys have used paired-end sequence reads to detect SVs in several individuals at high resolution [21-23], enabling identification at high confidence and subsequent analysis of hundreds of SV breakpoint sequences [21,22]. Several paired-end sequence read based methods have been described [21-25], some of which employ next-generation DNA sequencing. One such approach is high-resolution and massive paired-end mapping (PEM) [21]. Paired-end based approaches, including PEM, have several advantages over other SV-detection approaches. They allow SV-reconstruction at higher effective resolution than SNP genotyping-based algorithms and have a higher sensitivity than present microarray-based approaches, which are typically to some extent affected by cross-hybridization in repeat-rich regions. Furthermore, in contrast to SNP genotyping and microarray-based as well as read-depth-based approaches, they enable the identification of copy-number balanced SVs, such as inversions. Moreover, the comprehensive and high-resolution SV identification facilitated by PEM is more economical than assembly comparison or split read analysis. PEM is presently becoming more affordable due to the ongoing developments and cost decreases in next-generation DNA sequencing. Thus, PEM has recently been adopted for SV mapping in personal genomics endeavors such as the 1000 Genomes project and other personal human genome sequencing projects [23,26] as well as for the mapping of structural alterations in cancer tissues [16].

Thus far, paired-end sequence read-based surveys have mostly used custom approaches for SV detection, partially with *ad hoc *criteria. Although experimental validations indicated a reasonably successful performance of these approaches, a suitably parameterized approach to SV calling will be necessary to generate high confidence SV sets and to optimize the specificity and sensitivity of SV calling. In this regard it is evident that future studies that will utilize dense maps of structural variation in the genome for associating SV genotypes with phenotypic data will rely on high-confidence methods for SV calling. We thus developed a computational approach, *Paired-End Mapper *(*PEMer*), for mapping SVs at high resolution with a confidence measure and then analyzing them with a built-in database. Incorporated error models based on extensive simulations facilitated parameterization of *PEMer *and an evaluation of its performance. We benchmarked the computational approach on different datasets to show that it achieves SV assignments with improved sensitivity and specificity over previous paired-end sequence read based approaches for SV identification. *PEMer *can process data from several next-generation DNA sequencing platforms, for example, platforms from 454 (Roche), Illumina, and ABI. *PEMer *can be downloaded from [27], where instructions on how to install the framework are provided.

## Results

### Optimal computational detection of SVs using *PEMer*

**Figure 1:**
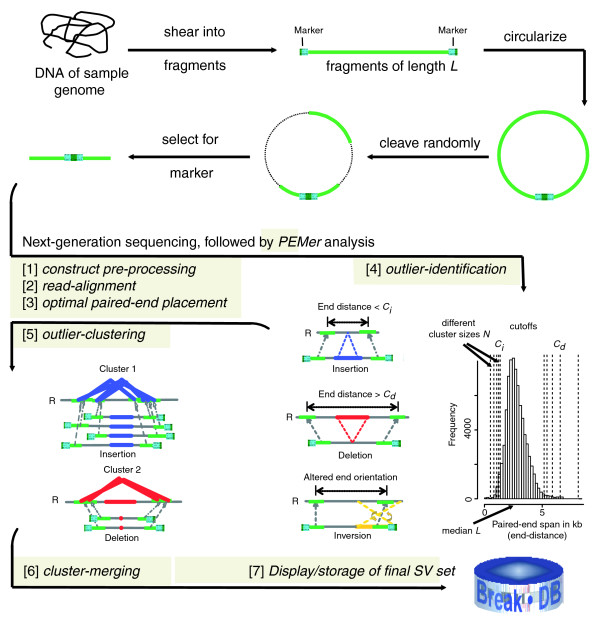
Scheme depicting computational steps carried out by *PEMer*. In PEM, when using the 454/Roche platform, randomly sheared genomic fragments are circularized and cleaved randomly into sequence stretches amenable to ultrafast sequencing (figure adapted and extended from Figure 1 in [21]). We subject resulting DNA sequences to *PEMer *for calling SVs relative to the reference genome ('R'). By default, *PEMer *uses the following processing steps: [1] construct pre-processing, [2] read-alignment, [3] optimal paired-end placement, [4] outlier-identification, [5] outlier-clustering, and [6] cluster-merging. Subsequently, [7] SVs (insertions, deletions, inversions, and more complex events) are displayed and stored in a back-end database for further analysis. In the outlier identification step, several different cutoff points *C*_*i *_and *C*_*d *_for the paired-end span, which are derived from the known insert-size distribution, are applied using a multi-cutoff strategy together with distinct minimally required paired-end cluster sizes *N*. After merging clusters constructed using different cutoff points, different PEM libraries, or different next-generation DNA sequencing platforms, an enhanced SV call resolution may be achieved.

The paired-end sequence reads based method PEM, as well as the underlying strategy used for scoring PEM data, are depicted schematically in Figure 1. In PEM, the end stretches of randomly picked genomic DNA fragments of an individual are sequenced and compared to a reference genome. For that purpose, initially, random genomic DNA fragments with a known and fairly tight size distribution are generated. For instance, the PEM protocol from 454/Roche involves hydrodynamic shearing resulting in a lognormal fragment length distribution centered at the median fragment length, or insert size, *L *(for example, with *L *= 2.5 kb; Figure 1). In PEM, indels relative to the reference genome are identified by relating the distance in base-pairs between the fragment ends mapped onto the reference genome (that is, the paired-end span) to the known insert size distribution (Figure 1). Furthermore, by comparing the relative orientations or positions of mapped ends inversions or more complex SV events (see Materials and methods) relative to the reference genome can be identified. Our approach *PEMer *uses an optimized pipeline for calling SVs from datasets generated by several different next-generation sequencing platforms. Therefore, *PEMer *implements a number of subsequent computational procedures, or steps, which have been developed as a set of modular components (described in detail in the Materials and methods section; see also Figure 1). First, in the 'construct pre-processing' step, the data are formatted into a proper structure. Second, in the 'read-alignment' step ends are first rapidly indexed against and then carefully aligned onto a reference genome. Third, pairs of mapped ends are combined into paired ends in the 'optimal paired-end placement' step. When processing relatively short sequenced ends (for example, such as those generated with the Solexa/Illumina or SOLiD/ABI platforms) we recommend novel read-indexing approaches that directly compensate for variation in the mappability of short sequences in the context of a complex, repeat-rich reference genome [28,29]. Fourth, in the 'outlier-identification' step outlier paired ends are recognized. Outliers are characterized by ends mapping onto the reference genome with a distance that is significantly deviating from expected paired-end spans (indicating an SV indel) or by ends matching onto different strands of a chromosome (indicating an inversion) or in different order relative to each other (potentially indicating a complex SV). Fifth, the 'outlier-clustering' step combines paired ends that likely originated from the same SV into clusters. Sixth, clusters obtained using different parameterizations - that is, by applying different cluster sizes and according cutoffs for outlier identification - are joined in the 'cluster-merging' step. Cluster-merging further enables combining data from different PEM libraries or from different next-generation sequencing platforms. This in turn helps increase the size range at which SVs can be detected and may add extra confidence in SV assignments through support from independent libraries or platforms.

**Figure 2:**
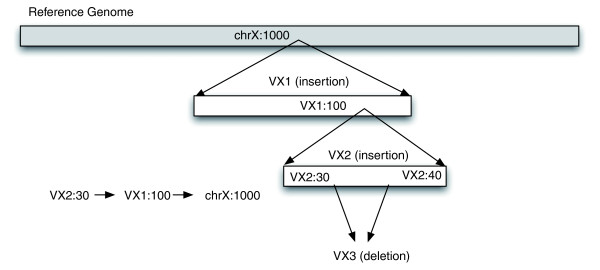
Depiction of the strategy used for assigning genomic coordinates to complex SV events. Coordinates within our database BreakDB are stored in a recursive fashion, if multiple SVs with partially overlapping coordinates occurred within a single haplotype. In particular, where a coordinate is typically defined with respect to the reference genome, it can also be defined in respect to other SVs, as indicated in the scheme depicted in the figure. For example, an insertion event can take place within an earlier insertion event, affecting the same haplotype the earlier event occurred in. If coordinates for the second insertion event were reported merely relative to the human reference genome, positional information for the SV would be lost. BreakDB therefore reports both coordinates within the ancestral ('parent') event, but can also trace back all the way to the reference coordinates.

Finally, *PEMer *reports the merged clusters, which can be displayed and stored. To facilitate the display, storage, and further analysis of variants, our approach contains a special database for handling SV data from various sources. The database, for which a schematic is depicted in Figure S1 in Additional data file 1, allows for a smooth connection between called SVs, clusters of outlier paired ends, and the underlying sequence reads. The database enables consideration of complex SV assignments from base-pair resolution data - different SVs may partially overlap in their genomic coordinates or they may be 'embedded' within each other. As such, they may have occurred as a consequence of subsequent, partially intersecting *de novo *events affecting the same haplotype. Accordingly, we developed a recursive data definition for SVs, in which the coordinates of a SV may be stored either with respect to the reference genome or with respect to one another (Figure 2).

### Parameterization and benchmarking of *PEMer *using simulations

It is critical to properly parameterize *PEMer *in order to optimize the specificity, sensitivity, and resolution of the approach. Since the highly non-uniform nature of the human genome causes difficulties in deriving a parameterization analytically, we chose to use simulations for estimating parameters and SV-calling efficiency. Namely, we placed sets of SVs into a known reference DNA sequence, and then used the modified genomic sequence to simulate PEM experiments. Specifically, simulations were carried out in the context of the general repetitive structure of the genome, with SVs randomly placed relative to highly repetitive elements and segmentally duplicated regions. In our simulations, we furthermore applied a realistic PEM fragment size distribution and a reasonable span coverage (that is, physical coverage, taking into account the amount of DNA sequence in the reference genome spanned by paired ends) expected to be sufficient for detecting most SVs. Instead of using the entire human reference genome, we performed the simulations on a diploid chromosome 2. (The euchromatic regions of chromosome 2 encompass approximately 8% of the genome; thus, simulations required relatively little computing time.) The genomic background was altered by randomly introducing a set of SVs of various sizes near the expected boundary of resolution of *PEMer*. For instance, we initially chose to generate sets of 100 heterozygous deletions, respectively, in sizes of 1, 2, 3, 4, 5, 6, and 10 kb. These are arbitrary, but suitable, SV set sizes enabling an evaluation of the sensitivity of *PEMer*. In addition, we also simulated different SV types. Finally, we simulated PEM data generated with different library insert size distributions and with different next-generation DNA sequencing platforms (see below).

Three essential parameters influence the performance of *PEMer*: the span coverage *λ *(which is proportional to the insert size *L*; see Materials and methods); the minimum number *N *of clustered outlier paired ends necessary for calling a SV; and the cutoff *C *for calling outliers. Based on the Poisson approximation, we initially estimated that for a diploid genome, a span-coverage *λ *of 4.75× will be minimally required to cover 95% of the heterozygous SVs within the detection range of PEM by at least two paired ends (see supplementary methods and notes in Additional data file 1). For simplicity, we applied a rounded *λ *= 5× in most analyses below. We then used *PEMer *to reconstruct SVs in the simulated genomic DNA and evaluated its performance by applying various values for *C *and *N*. We generally applied three distinct strategies for SV identification.

#### Strategy one

The 'single cutoff' strategy was implemented as the previously most widely applied scoring approach for identifying SVs from PEM data (for example, described in [30]). The single cutoff applies a fixed required cluster size *N *of 2 and regards paired ends as outliers if the measured paired-end span exceeds a certain cutoff *C*, which is typically set at 3 standard deviations from the median (the median usually can be interchanged with the mean; note, for example, that in case of the 454/Roche platform the median is essentially identical to the mean in log-space). All outlier clusters of size *N *= 2 or larger are considered as SVs, whereas unclustered outliers are discarded. The cluster-merging step is unnecessary when applying this strategy.

#### Strategy two

**Figure 3:**
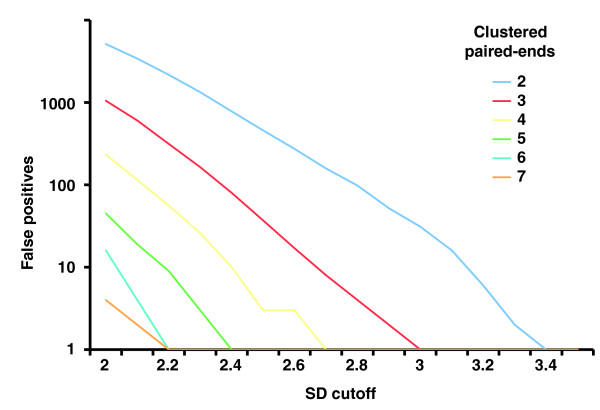
Numbers of false positive SV calls in relation to the cutoff used for defining outliers. Cutoff values for defining outlier paired ends are given in terms of standard deviations (SDs) from the median of the expected distribution of paired-end spans (which in turn is derived from the insert size). PEM data generated with the 454/Roche platform were simulated applying a median insert size *L *= 2.5 kb and a span-coverage of *λ *= 5× of the diploid chromosome 2. To arrive at *λ *= 5×, only optimally (uniquely) placed paired ends were considered when estimating *λ *('effective span coverage'). Here, the genome-wide count of false positives is put in relation to outlier-identification cutoffs for various required cluster sizes *N *('clustered paired ends') of 2 up to 7. 'False positives' refers to the number of false positives identified on chromosome 2.

In the 'multi-cutoff' strategy different cluster sizes *N *from 2 to 6 were applied together with different corresponding cutoffs *C *for outlier identification (Table S1 in Additional data file 1 and Figure 3). Note that, in theory, *N *= *∞ *represents the limit; however, in reality at *λ *= 5× we did not observe additional SV calls when setting *N *to values greater than 6. The multi-cutoff strategy enables an enhanced resolution compared to the single cutoff strategy. In this regard, for a given cluster size *N *we conservatively defined the optimal cutoff *C *as the one for which no false positives and a maximum possible number of reconstructed SVs were observed in our simulations.

#### Strategy three

The 'simplified multi-cutoff' was implemented as a compromise between the single cutoff and the multi-cutoff, using only cluster sizes *N *of 2, 3 and 4. During our simulations, results for the simplified multi-cutoff were nearly identical to the multi-cutoff strategy (Table 1), but had the benefit of a decreased computing time (see below).

**Table 1:** Results of simulations indicating the reconstruction efficiency of *PEMer *for heterozygous deletions of different sizes

SV size	Single cutoff	Multi-cutoff	Simplified multi-cutoff	Multi-cutoff*	Simplified multi-cutoff*
1000	3 (4)	3 (4)	3 (4)	3 (4)	3 (4)
2000	12 (13)	23 (26)	21 (23)	11 (13)	6 (6)
3000	52 (57)	61 (68)	61 (68)	49 (52)	44 (46)
4000	84 (85)	85 (86)	85 (86)	80 (82)	80 (82)
5000	91 (93)	91 (93)	91 (93)	91 (93)	91 (93)
6000	92 (92)	92 (92)	92 (92)	92 (92)	92 (92)
10000	88 (91)	88 (91)	88 (91)	88 (91)	88 (91)

Total	422 (435)	443 (460)	441 (457)	414 (427)	404 (414)
False positives (chromosome 2)	31 (31)	31 (31)	26 (31)	5 (4)	2 (1)

Each row displays statistics for reconstructed heterozygous deletions of a particular size, derived from simulations of 454/Roche based PEM data. Columns show numbers of identified SVs for each reconstruction strategy. Numbers in parentheses correspond to simulated SV reconstructions without sequencing error. All SVs were reconstructed at an effective span coverage *λ *= 5× (where, *λ *was assessed after optimal paired-end placement) of a simulated diploid chromosome 2. Note that for the single cutoff strategy events ≥ 5 kb were reconstructed at a level near the theoretical maximum of 95% derived from the Poisson approximation (see supplementary methods and notes in Additional data file 1). However, the multi-cutoff and simplified multi-cutoff strategies outperformed the single cutoff strategy in detecting SVs < 4 kb. *We also applied alternative - that is, optimal - cutoff parameters, for which the sensitivity is similar to the single cutoff, but for which a false positive rate of approximately 5% was observed.

We initially assessed the SV-reconstruction capability of *PEMer *for heterozygous deletions by simulating data from the 454/Roche platform, and observed that the single cutoff is efficient and sufficient for the reconstruction of deletions of 4-5 kb or longer (Table 1). However, the multi-cutoff strategy was clearly superior for reconstructing SVs smaller than 4 kb. When applying both strategies with a realistic simulation-based sequencing error (see below) and with parameters resulting in similar false positive call rates, respectively, 30% additional events smaller than 4 kb and 73% additional events smaller or equal to 2 kb were identified with the multi-cutoff compared to the single cutoff, whereas the reconstruction efficiency for events >4 kb was similar among both strategies. Interestingly, the simplified multi-cutoff achieved results that are practically the same as for the multi-cutoff (Table 1) with a decreased computing time. This suggests the existence of a boundary on the optimal cluster size *N *at a given span coverage.

False positives were recorded during the simulations as SV calls of any type (deletion, insertion, inversion, or complex) generated by outlier paired ends not resulting from a simulated SV. As we describe in Additional data file 1, we further monitored the generation of false positive calls from chimeric PEM library inserts [21] and found that the effect of such chimera on the false positive rate is negligible. Furthermore, we determined the expected genome-wide number of false positives by scaling the observed number of false positives with the factor 'size of the diploid genome divided by the size of the diploid chromosome 2'. We also derived an analytical formula for calculating numbers of expected false positive deletions and insertions (see Materials and methods) and validated the formula by comparison with the simulation-based results. This enabled us to calculate *E*-values and *P*-values for both SV types (Table S2 in Additional data file 1 and Materials and methods). We defined as the false positive rate the number of detected false positives scaled by the number of SVs that we expect to be ascertainable with paired-end sequence-based approaches operating at the size range of PEM - for example, approximately a thousand when using the 454/Roche platform [21]. Using conservative cutoffs expected to result in a false positive rate of approximately 5%, when applying 1,000 as the scaling factor, *PEMer *reconstructed approximately 90% of all simulated heterozygous deletions >4 kb with *λ *= 5× (see Results for all three strategies in Table 1), that is, approximately 95% of the SVs expected to be ascertainable (when relating the observed 90% to the 95% of events expected to be ascertainable at *λ *= 5×; see supplementary methods and notes in Additional data file 1). The rate of false positives can be reduced to near zero by applying more stringent cutoffs, which leads to a slightly diminished reconstruction efficiency (Table S3 in Additional data file 1).

Furthermore, we also analyzed heterozygous inversions and insertions by simulation. Specifically, we found that at 5× span coverage heterozygous inversions can be recovered with high reconstruction efficiency (>95%; Table S4 in Additional data file 1) and highly significant *E*-values (based on simulations; Table S2 in Additional data file 1). On the other hand, heterozygous insertions were reconstructed with poor efficiency (<10%) and at a small size-range when using a 2.5 kb insert size (Table S5 in Additional data file 1). Note, however, that when using a larger insert size of 10 kb, we observed a marked improvement of the reconstruction efficiency for insertions - to up to 70% - without reduction in the portion of reconstructed deletions (Table S6 in Additional data file 1). Note further that with a large insert size PEM becomes more cost-efficient, as longer DNA stretches are covered per sequenced base-pair.

We would like to stress that in most of our simulations, we conservatively assumed heterozygosity of SVs - that is, we assumed single instances of SVs per diploid genome. However, a large portion of SVs is homozygous [21,22]. As homozygous SVs display two instances per diploid chromosome set, they are usually covered by more paired ends and, thus, are more easily ascertainable than heterozygous SVs. To exemplify the higher sensitivity of PEM towards homozygous SVs, we simulated the reconstruction of homozygous deletions (Table S7 in Additional data file 1). Specifically, at a false positive rate of approximately 5%, more than 97% of the simulated homozygous deletions >4 kb were identified with *λ *= 5×. Furthermore, we observed an increased sensitivity in detecting SVs <4 kb (compare, for example, Table 1 and Table S7 in Additional data file 1). Finally, owing to the higher frequency at which homozygous SVs tend to be spanned by paired ends relative to heterozygous SVs, homozygous SVs are usually reconstructed with more highly significant *E*-values (see Table S2 in Additional data file 1; i.e. more highly significant *E*-values are achieved for SVs with a high number of spanned paired ends).

Finally, thus far we have focused on simulations of data from the 454/Roche next-generation sequencing platform. In the past months, PEM protocols have been developed by short-read-based next-generation sequencing platforms, including the Solexa/Illumina as well as the SOLiD/ABI platform. In order to assess the SV reconstruction efficiency for PEM data produced with a short read generating platform, we examined the SV-mapping capabilities of the Solexa/Illumina platform at 5× span coverage. Specifically, we applied a realistic paired-end insert size distribution centered at 250 bp and reasonable cutoffs for outlier identification (Table S8 in Additional data file 1) and observed a reconstruction efficiency for heterozygous deletions that is comparable to the rate at which SVs are identified by the 454/Roche platform (Table S9 in Additional data file 1).

### Sensitivity to sequencing errors

We also investigated the effect of sequencing errors on SV calling by reconstructing SVs using two sets of reads, with and without sequencing errors, introduced at a rate reflective of the respective next-generation sequencing platform. To this end, we have included specific error models for different sequencing platforms in our simulations (see Materials and methods). Notably, when testing the effect of sequencing errors on data from the 454/Roche platform, we found that sequencing errors only slightly affected the effective span coverage of PEM by decreasing it by 1.3%. Interestingly, when assessing the reconstruction efficiency using heterozygous deletions as an example, we found that sequencing errors had a negligible effect on SV calling for most SV sizes (Table 1). Nevertheless, a somewhat more pronounced effect was observed for short (<5,000 bp) SVs, for which, in general, more reads were required to enable SV assignments. Thus, SVs with a size at the margin of *PEMer*'s detection range appear generally (slightly) more sensitive to sequencing errors. We further observed that sequencing errors at a level typically occurring in 454 Sequencing have little influence on the overall false positive rate (Table 1).

**Figure 4:**
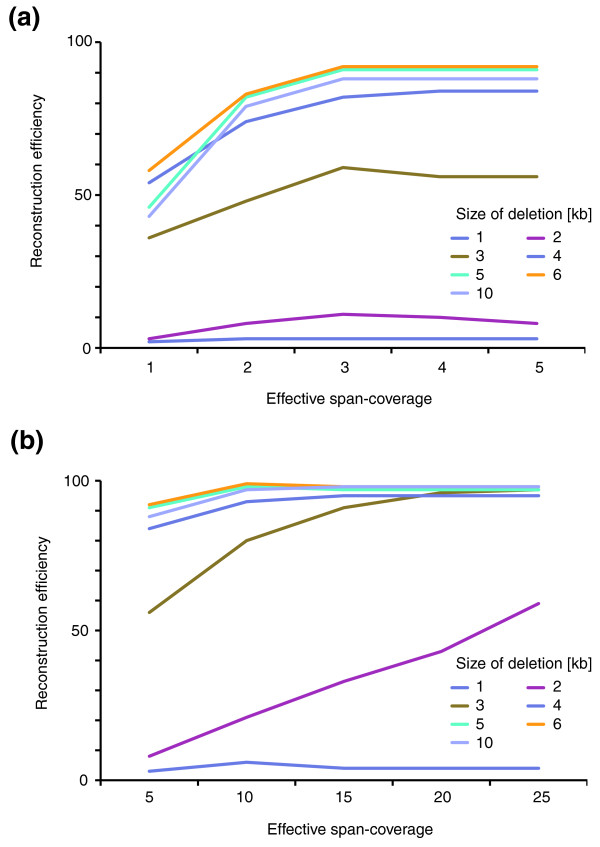
SV reconstruction efficiency in relation to the span coverage *λ*. We used simulations to generate heterozygous deletions 1-10 kb in size (median PEM insert size 2.5 kb; 454/Roche platform). Paired-end data were simulated with different span coverages *λ*; only optimally (uniquely) placed paired ends were considered when estimating *λ*. **(a) **Reconstruction efficiency for values of *λ *from 1× to 5×. **(b) **Reconstruction efficiency for values of *λ *from 5× to 25×.

Some genome studies analyze genomes at a span coverage considerably higher than 5×, with values of *λ *at 25× or higher. When expanding our simulations to allow for parameterization of *PEMer *at *λ *= 25× (Table S1 in Additional data file 1), we found that at such a span coverage deletions down to 3 kb are efficiently reconstructed in PEM datasets generated by the 454/Roche platform: in particular, 97% of all SVs of 3 kb in size are called at *λ *= 25×, whereas only 49% are called at 5× (Table S3 in Additional data file 1; Figure 4). Thus, the sensitivity in detecting smaller deletions generally increases significantly at high span coverage. We note that when using high span coverages, generally large values of *N *should be used. For example, at *λ *= 25×, *N *= 5 represents a suitable minimum cluster size (Table S1 in Additional data file 1), whereas smaller values of *N *lead to numerous false positives. Thus, notable gains in sensitivity and resolution can be achieved at high span coverage at the cost of a linear increase in sequencing costs.

### Modular design, alignment algorithms, and time complexity of *PEMer*

The sheer complexity and size of next-generation sequencing data sets impose challenges on procedures applied for mapping, storage, and analysis of the data [31], particularly in the light of novel ongoing large-scale human genome sequencing projects (for example, the 1000 Genomes project). Thus, we have put a lot of effort into optimizing *PEMer*, and carefully evaluated its time complexity. In particular, we found that the run time scales approximately linearly with the number of reads. Furthermore, the approach can be easily parallelized by processing bundles of sequencing reads on separate nodes of a computing cluster. If a genome as large and complex as the human genome is analyzed, read-alignment represents the time-limiting step of *PEMer*, taking approximately two-thirds of the computing time. To take this into account and to increase the flexibility of *PEMer *for next-generation sequencing technologies, *PEMer *has been developed in a highly modular fashion. For example, 454 Sequencing data can be rapidly mapped against the genome using two alternative indexing algorithms, that is, Megablast [32] or BLAT [33]; then high-quality alignments are constructed using the Smith-Waterman algorithm (see Materials and methods). By default, Megablast is used for indexing 454/Roche data, since we found Megablast to be slightly more sensitive than BLAT when using several parameter sets, albeit at the cost of a slight increase in computing time (Table S10 in Additional data file 1). On the other hand, Solexa/Illumina and SOLiD/ABI data are by default indexed using the fast MAQ algorithm [28] (see Materials and methods).

To exemplify the applicability of *PEMer *for processing large datasets we recorded basic timing data in the course of processing PEM data within the 1000 Genomes project (Table S11 in Additional data file 1). Specifically, *PEMer *required approximately 28,000 CPU hours for processing 74 million paired-end reads generated with the 454/Roche platform using a median fragment size of 2.5 kb. In this case, a large fraction of 454/Roche-specific linker sequences had already been mapped prior to *PEMer *analysis, leading to a considerable decrease in computing time, as linker-mapping is responsible for approximately a quarter of the overall computing time. To achieve a realistic estimate, we thus only considered the previously unmapped reads and estimated that 16,000 CPU hours would be required to map 10 million reads, the equivalent of approximately 4.5× span coverage of a diploid human genome. On a large-scale computing cluster with approximately 400 CPUs, application of *PEMer *to map SVs in a single individual is thus normally completed in approximately 2 days.

### Benchmarking *PEMer *on previously published paired-end datasets

Finally, we applied *PEMer *on previously published paired-end datasets [21] from a presumably European female (NA15510) and an African female (NA18505) to evaluate whether the approach indeed allows for an increased efficiency in SV calling. We therefore estimated optimized cutoffs and applied the simplified multi-cutoff strategy to search for additional SVs not previously reported using a stringent cutoff expected to result in 0% false positives (based on simulations). Our analysis revealed 18 SV indel events overlooked previously [21], which are summarized in Table S12 in Additional data file 1: that is, 16 in NA18505 and 2 in NA15510, an individual that had been sequenced at relatively low (approximately 2×) span coverage. We analyzed all novel SVs manually on the UCSC browser, and found that out of these 18, 15 (83%) overlapped with previously identified SVs listed in the Database of Genomic Variants [34]. Furthermore, the high resolution of our SV calls allowed us to infer plausible SV formation mechanisms for 11 (61%) SVs (Table S12 in Additional data file 1), including all three that did not intersect with variants listed in the Database of Genomic Variants. In particular, we inferred that five SVs were likely formed by retrotransposition [35]. Furthermore, in six instances satellite DNA expansions appear to have caused SV formation. For the remaining SVs we were not able to discriminate between possible formation mechanisms [21,22] owing to the lack of high-resolution breakpoint data. Furthermore, as expected, all novel SV calls were near the expected boundary of resolution of *PEMer*. This led, for example, to an increase of 30% in the rate at which deletions <4 kb were detected compared to a previous study using the 454/Roche platform [21], indicating a gain in sensitivity and resolution at the margin of previous PEM-based scoring approaches.

## Discussion

We have developed a computational approach, *PEMer*, which facilitates the identification of SVs from large-scale PEM data. *PEMer *enables processing data from several widely applied next-generation sequencing platforms. We parameterized *PEMer *with a newly developed simulation framework, and demonstrated using simulations and real datasets that this results in an improved SV-calling performance at the margin of previous paired-end-based approaches. Thus far, surveys mapping small SVs systematically across several individuals have been lacking despite an abundance of SVs at this size range. In particular, when re-scoring a recently published dataset with *PEMer*, we were able to report 18 additional SVs beyond the detection range of previous computational approaches for scoring PEM data. We provided independent evidence for all 18 SVs using data-mining and sequence analysis, suggesting a low false positive rate in SV calls.

We note that the herein described simulations were carried out using reasonable parameter settings. Realizing the utility of simulations to parameterize SV-calling methods, we decided to make available our simulation software to the community in conjunction with *PEMer*. We realize that our simulation software may also be useful in distinct contexts where paired ends are being used successfully, such as for transcript analysis [36,37] or the detection of gene fusions caused by recurrent translocations in cancer [38].

Our study also has certain limits, as discussed below.

### Segmental duplications

With regard to the simulations, we did not specifically generate SVs with breakpoints embedded in long stretches of repetitive sequence - such as segmental duplications that may induce SV formation through non-allelic homologous recombination. Specifically, a portion of SVs formed through non-allelic homologous recombination is likely to be overlooked by *PEMer *due to the relatively short length of sequenced end stretches (for example, approximately 110 bp for the 454/Roche platform and <40 bp for the Solexa/Illumina or SOLiD/ABI platforms), which hampers unambiguous genomic alignments. In this regard, note that all presently available SV-detection approaches (including microarray-based approaches) are limited in terms of detecting SVs embedded in segmental duplications, and that the true extent of such SVs is thus unknown.

### Single nucleotide polymorphisms

The simulations currently do not consider the presence of SNPs in the sample genome. Similar to base-calling errors, SNPs, which, on average, affect 1 in a 1,000 bases, may lead to read misalignment, particularly in repetitive regions with diminished mappability such as segmental duplications. Note, however, that the catalogue of known SNPs is presently incomplete in these genomic regions. Note further that the optimal paired-end placement step can, to some extent, compensate for both base-calling and SNP-based misalignment errors (see supplementary methods and notes in Additional data file 1).

### Insertions versus deletions

Due to the insert size distributions commonly used in PEM, the size range at which insertions can be identified is considerably smaller than for deletions and inversions. Particularly, large insertions (for example, events ≥ 3 kb when using a median insert size of 2.5 kb) may be missed when merely analyzing significant deviations from the mean paired-end span. Note that this problem can, in part, be compensated for by selecting a range of insert size distributions (see, for example, Table S6 in Additional data file 1) and by reconstructing large SVs as mated insertions [21]. For personal-genomics efforts such as the 1000 Genomes project it will thus make sense to generate more than one paired-end library per sample, with one library optimally involving a relatively large insert size (that is, 10 kb or larger). Note that these libraries can be analyzed at fairly low additional costs, as relatively small numbers of paired ends are required to achieve sufficient span coverage when using large insert sizes.

### Genome expectation statistics

We estimated the likelihood for covering a genomic element using the Poisson-approximation, assuming that SVs and paired ends are uniformly distributed in the genome. Furthermore, our simulations also assumed a uniform distribution of SVs in the genome. We realize that, in the future, concepts applied in this study may be extended by using more sophisticated models of genome expectation statistics such as the 'genome structure correction' used in the recently published Encode consortium paper [39], which considers the distribution of gaps, repeats, and SVs in the reference genome.

Specifically in relation to present limits, we would like to emphasize the design of *PEMer *as a modular tool, for which specific parts can be fairly easily optimized and improved. Examples for possible improvements include the consideration of novel approaches for compensating for the variation in the mappabilty of reads within the reference genome [29], and an improvement of the overall computing time when processing large datasets. For example, in relation to the current read-alignment step, we realize that a considerable amount of time may be saved by applying novel, time-efficient sequence alignment approaches geared towards the specific read lengths applied in the study.

Interestingly, the influence of sequencing errors on SV calling is minor - for example, when compared to the influence of base-calling errors on SNP-assignments. In the future, next-generation sequencing technologies that allow for longer DNA sequence reads than presently feasible will increase the sensitivity of *PEMer *in repeat-rich regions by ensuring that ends are mapped onto the correct location in the genome.

Lastly, while our paper was in preparation, Lee *et al. *[40] published an alternative approach for SV detection based on paired-end sequence reads. In contrast to *PEMer*, the approach by Lee *et al. *has been developed for processing Sanger dideoxy sequencing reads, rather than next-generation sequencing reads. While it is likely that both approaches or concepts thereof will be applied for SV detection in the future, a preliminary comparison of both approaches indicates a higher overlap with previously reported SVs for *PEMer *calls compared to calls by the Lee *et al. *approach (see supplementary methods and notes in Additional data file 1). One possible explanation for this observation may be a higher specificity of SV calls generated by *PEMer *compared to the approach by Lee and colleagues.

In conclusion, *PEMer *facilitates SV detection from large-scale next-generation DNA sequencing datasets on a normal computing cluster. We would like to point out that early versions of *PEMer *have already been used extensively in studies focusing on several individual genomes (1000 Genomes project and [21]). Recognizing the increased usage of paired-end sequencing technologies for personal genomics [23,26] and for high-resolution SV surveys [21,22], we decided to make the code of *PEMer*, together with executables and a proper documentation, available to the community over the world-wide web.

## Materials and methods

### Components and modules included in *PEMer*

*PEMer *consists of the following modular components, which are by default executed in the order given below.

#### Construct pre-processing

Initially, PEM data are formatted into a proper structure. For example, when processing data from the 454/Roche platform, the standard 44 bp linker sequence (GTTGGAACCGAAAGGGTTTGAATTCAAACCCTTTCGGTTCCAAC) from the 454/Roche paired-end protocol is identified (for example, at a minimum sequence identity of 90%) and fragments split into paired ends using the linker as a seed. PEM data generated with the Solexa/Illumina or SOLID/ABI platforms are pre-processed and initially aligned to the reference genome using MAQ [28].

#### Read-alignment

In this step, both ends are independently aligned with the reference genome. When using 454 data, by default, a computational approach that combines efficient initial heuristic genome alignment (that is, using Megablast [32] by default with parameters: '-p 80 -s 11 -W 11', or, alternatively, using BLAT [33] with parameters '-fastmap') and comprehensive optimal realignment (that is, using the Smith-Waterman algorithm [41]) is used. As mentioned above, MAQ [28] (default parameters) is normally used for processing Solexa/Illumina or SOLiD/ABI data. In principle, any sequence alignment algorithm can be plugged into *PEMer *for read alignment.

#### Optimal paired-end placement

In this step, when processing data from the 454/Roche platform, an adapted version of the placement algorithm [30] is implemented to enable the identification of most plausible paired-end alignments. This is particularly important in cases where alignments are ambiguous due to the repetitive nature of the human genome. In brief, the placement algorithm executes a cost function that penalizes outlier paired-end assignments, if one or both ends display high sequence similarity to a different genomic locus and if placing the end(s) into the alternative locus would result in a non-outlier paired end (see supplementary methods and notes in Additional data file 1). When processing Solexa/Illumina or SOLiD/ABI paired-end data, *PEMer *by default omits the abovementioned placement algorithm, and instead considers paired ends as optimally placed if each end unambiguously aligns against the reference genome with a MAQ [28] 'mapping quality' of at least 20. This score-cutoff [26] ensures unambiguous optimal placement of short reads onto the reference genome.

#### Outlier identification

Paired ends are considered as outliers if they map with a relative orientation of ends, or genomic position, consistent with structurally altered genomic regions - for example, if they fall outside the expected range of paired-end spans. Paired ends falling beyond the expected range of spans are identified based on a cutoff *C *expressed in terms of standard deviations from the median *L *and the according cutoff points *C*_*i *_and *C*_*d *_(Figure 1). The cutoff points are usually derived from simulations and depend on the span coverage *λ*, the cluster size *N*, and the distribution of paired-end spans. Alternatively, the cutoff points may be derived from experimental controls, or may be estimated directly from the sample data. Note that *PEMer *by default discards outlier paired ends in which both ends map to different chromosomes (that is, putative translocations).

#### Outlier clustering

Outliers are categorized into SVs if a cluster of *N *(or more) independent paired ends is consistent with a single SV. *PEMer *evaluates whether all paired ends in a cluster are indicative of the same event. In other words, a simple intersection of paired ends may be insufficient - for example, if two intersecting paired ends indicate deletions with significantly different predicted deletion sizes. (Thus, a window for the proper clustering of paired ends is defined in *PEMer*, as described below in the section 'Estimating *E*-values and *P*-values'.) Deletions are identified from ≥ *N *overlapping discordant paired ends with a paired-end span >*C*_*d *_(with the condition that both putative breakpoints are spanned). Insertions may be identified from ≥ *N *overlapping discordant paired ends exhibiting a paired-end span <*C*_*i*_. Inversions are identified using ≥ *N *paired ends that are discordant in terms of orientation relative to the reference genome and are consistent with a single inversion breakpoint - that is, in such a way that all paired ends span a single, common breakpoint interval. In addition to those simple SV events, more complex events [21] may be identified by *PEMer*: mated insertions are identified from ≥ *N *unpaired SVs that lie in nearby genomic regions and have ≥ *N *paired ends indicating a connection with a (common) distal genomic region <100 kb in size. Mated insertions may involve tandem duplications, or translocations. Unmated insertions are predicted from ≥ *N *paired ends that support a rearrangement of a genomic region in which loci change relative order without changing their relative orientation (that is, both ends map to the same DNA strand).

#### Cluster merging

Clusters consistent with the same SV are merged into a single cluster. This step is necessary when SVs are searched in parallel with distinct cutoffs and cluster sizes (for example, when using the multi-cutoff or simplified multi-cutoff strategy, or when results from paired-end datasets generated with different insert sizes or different next-generation sequencing platforms are combined).

### Simulation of PEM experiments

We performed simulations with a diploid chromosome, which enabled evaluation of both the efficiency of SV reconstruction and the false positive rate of *PEMer*. In particular, we used human chromosome 2, the chromosome with the largest determined length as well as an average repeat content and gene density. While we regard the selection of chromosome 2 as a reasonable pick to save computational processing time during the simulations, future studies may use other chromosomes or entire genomes in haploid or diploid form to parameterize *PEMer*. SV events were randomly generated, that is, distributed uniformly on the chromosome as described above. When simulating the reconstruction of heterozygous SVs, no events were introduced on the second copy of chromosome 2, which was included only to monitor the false positive rate. For example, when simulating PEM data generated by the 454/Roche platform, first, random shearing of the sample genome was carried out by randomly picking DNA fragment lengths from a given lognormal distribution with reasonable values for median (that is, 7.8 in log-space) and standard deviation (0.29 in log-space), which both were obtained from a typical PEM experiment. Second, fragment centers were uniformly placed along the chromosomes. Third, DNA fragment circularization, random cleavage and linker read isolation were simulated by first generating read lengths from the length distribution of sequences resulting from a typical 454 run (that is, the Roche GS-FLX-system), then by placing 'centers' for the 44 bp linker sequence uniformly onto the read, then by placing the linker sequence onto that center, and, finally, by assigning sequences of DNA fragment ends to the read ends not occupied by the linker. To achieve the expected topology of paired ends in the circulated DNA (Figure 1) the 5'-end of the fragment was assigned to the 3'-end of the read and vice versa. Fourth, the resulting fragments, which were in principle undistinguishable from real genomic fragments generated by the PEM method indicated in Figure 1, were subjected to *PEMer *for SV detection.

Finally, simulation parameters can be easily adapted to platforms generating short PEM end tags (see Tables S8 and S9 in Additional data file 1 for an example involving the simulation of Solexa/Illumina data).

### Error models for next-generation DNA sequencing

Optionally, error models may be applied in our simulations to consider typical next-generation sequencing errors. For example, our simulations enable considering the major two causes of errors in 454 Sequencing, that is, insertion of nucleotides and homopolymer errors. In the error model we assumed that signals observed from a homopolymer of length *n *follow a Gaussian distribution with mean *n *and the standard deviation being proportional to the square root of *n *with coefficient 0.17, while the background follows a lognormal distribution with mean 0.2 and standard deviation 0.1 (Figure S2 in Additional data file 1) [42,43]. We used intersection points of the curves as cutoff points for calling a particular DNA sequence for a given signal. For instance, signals in the range 0.56 to 1.43 were called as a single sequenced nucleotide (Figure S2 in Additional data file 1), rather than a homopolymer (that is, dimer). Nucleotide flow was simulated in the following order: T, A, C, G. For every nucleotide sequenced (including homopolymers and single nucleotides) the observed signal was generated either from a background distribution - in cases where the flowed nucleotide was different from the nucleotide to be sequenced - or otherwise from the corresponding Gaussian distribution. The overall sequencing error rate was 2.5%.

For the Solexa sequencing error we approximated the average substitution rate of the Solexa/Illumina platform [44] using a simple model involving a fourth degree polynomial. The polynomial was used to assign substitution probabilities at each base position during the simulation. If at a given sequenced position a substitution was assigned by the simulation procedure, a randomly picked, different nucleotide was inserted at the position in question. The average sequencing error rate was 1.5%.

While our simulations were designed for optimizing the parameters of *PEMer *and, thus, for improving the resolution over earlier approaches, we realize that future studies may aim for broader and more realistic simulations of PEM-based studies. To facilitate future simulations involving PEM data generation and scoring, we have made the code of our simulation scripts available to the public together with *PEMer*.

### Development of a specialized breakpoint database

To allow storage, display and manipulation of SV data as well as consistency between different sets of SVs, we implemented a database module for our approach. In particular, a web-accessible database, BreakDB, was developed, which holds a variety of data along with each SV entry. A diagram of the BreakDB schematic, illustrating the database tables and their relationships, is depicted in Figure S1 in Additional data file 1. Data inserted into BreakDB can easily be manipulated for subsequent analyses of the SV data - for instance, high-resolution breakpoint information (i.e. the genomic coordinates of breakpoint-junctions), the expected overall coverage of SVs in a particular genome estimated based on the Poisson approximation [45] (see Additional data file 1), and results of breakpoint junction analyses by BLASTN [46] can be added to a SV entry and mined once becoming available. Therefore, the database has a versioning system, so that all changes to an event are archived and are viewable within the application. BreakDB contains information such as the coordinates, flanking and inserted sequences (in case breakpoints are known), potentially the suggested molecular mechanism leading to SV formation, and supporting evidence for the SV entry. With more SVs identified at base-pair resolution, their representation in databases becomes challenging as the coordinates of independently occurred SV events that subsequently affected the same locus may overlap in a complex fashion. To deal with such scenarios, subsequent SV events - for example, an insertion of genomic DNA followed by inversion and deletion of parts of the sequence - can be defined recursively in BreakDB (Figure 2). Thus, a SV event can be defined with respect to the current version of the reference genome (build36), or, in case of complex embedded SVs, with respect to another SV. Periodically, as a SV collection becomes stabilized, a release is generated and displayed as consistent, static pages.

### Estimating *E*-values and *P*-values

*PEMer *computes *E*-values and *P*-values for the different types of SVs identified in PEM datasets. Specifically, given a certain span coverage we can estimate the total number of optimally placed paired ends. Let us assume the span coverage is calculated as *λ *= *NL*/*G*, where *N *= number of optimally placed paired ends, *L *= median insert size, and *G *= diploid genome size; thus, rearranging *N *= *λG */*L*. Now, let us introduce a set *Y *of discordant paired ends with *N*_*h *_elements and a span beyond a cutoff *H *indicative of deletions, that is, all paired ends with a span larger than a cutoff expressed in terms of standard deviations *SD *from the mean of the distribution of spans. Note that as for *H *> 2 × *SD *the frequency of occurrence of the span lengths decreases faster than exponentially, effectively all spans in the set *Y *are approximately of length *H*.

Consider consequently placing *N*_*h *_paired ends randomly onto the genome and checking whether the *n*-th newly placed paired end forms a proper cluster with the already placed *n-1 *paired ends. The probability to cluster will be:

*P*(*n*) =(*n-1*)*W*_*h*_/(*0.5 G*)

where *W*_*h *_is the effective window size applied for clustering long paired ends (i.e., paired-ends indicative of deletions), (*n-1*)*W*_*h *_represents the number of nucleotides where the *n*-th paired end may fall in order to form a proper cluster with any previously placed paired end, and *0.5 G *is the non-redundant length of the genome (that is, the size of the haploid genome). Note that since all paired ends are effectively of given size *H*, *W*_*h*_/*2 *is simply the maximum separation between the ends of two pairs so that that they still cluster. We further assume that *Y *covers the genome sparsely, thereby neglecting the effect of window overlap from different paired ends. Thus, the total number of clusters will be:

$$E_{h}=\sum_{n=1}^{N_{h}}P(n)=N_{h}(N_{h}-1)\frac{W_{h}}{G}$$

where *E*_*h *_is the expected number of false positive deletions, or an *E*-value in relation to the number of randomly clustered 'long' discordant paired ends of set *Y*. Similarly, one can generalize the equation for calculating the number of false positives for *k *(2 or more) overlapping paired ends:

$$E_{h}=\frac{N_{h}!}{k!(N_{h}-k)!}{\left(\frac{2W_{h}}{G}\right)}^{k-1}$$

By using similar reasoning, one can estimate that the expected number *E*_*s *_of false positive insertions is:

$$E_{s}=\frac{N_{s}!}{k!(N_{s}-k)!}{\left(\frac{2W_{s}}{G}\right)}^{k-1}$$

where *N*_*s *_is the number of 'short' discordant paired ends at cutoff *S *indicative of insertions and *W*_*s *_is the effective window size applied for clustering short paired ends (i.e., such indicative of insertions).

*PEMer *detects SVs by clustering long and short events separately and is flexible in terms of defining *W*_*h *_and *W*_*s*_, which are used to cluster paired ends based on compatibility in sizes and locations of ends. Thus, the values *W*_*h *_and *W*_*s *_are introduced to simplify the clustering procedure and to obtain an analytical description of the false positive rate. We estimated that when clustering two paired ends the window sizes correspond roughly to the cutoff used for clustering *W*_*h *_~*H *and *W*_*s*_~ *S*. By comparison with simulated data we also found that the effective *W*_*h *_doubles when tripling the minimum required number of paired ends in a cluster while *W*_*s *_gets twice longer when doubling the minimum required number of paired ends in a cluster; therefore, we obtain the general dependency *W*_*h*_~ (*k *+ 1)*H*/3 and *W*_*s *_~ *kS*/2. Thus, the amount of false positives is estimated as:

$$E_{h}\approx \frac{N_{h}^{k}}{k!}{\left(\frac{2\left(k+1\right)H}{3G}\right)}^{k-1}$$

and

$$E_{s}\approx \frac{N_{s}^{k}}{k!}{\left(\frac{kS}{G}\right)}^{k-1}$$

In order to calculate *P*-values (*P*; the chance that a given event with a given cutoff occurred at random), we need to convert the number of randomly generated events *E *into the probability that an event will happen at least once. Assuming the number of false positives in a given experiment follows a Poisson distribution with mean *E*, we can use the known analytical expression *P *= 1 - exp(-*E*), with $E\approx \frac{N_{q}^{k}}{k!}{\left(\frac{2\left(k+1\right)Q}{3G}\right)}^{k-1}$ for clusters of 'long' paired ends and $E\approx \frac{N_{q}^{k}}{k!}{\left(\frac{kQ}{G}\right)}^{k-1}$ for clusters of 'short' paired ends. Here, *N*_*q *_is the quantile of events corresponding to the 'shortest'/'longest' paired end of length *Q *in the cluster defining a deletion/insertion.

Examples of *E*-values for identified SVs are given in Table S2 in Additional data file 1. We usually operate with *E*-values rather than with *P*-values, as they can be more intuitively understood. For *E *< 0.01, *P *and *E *are nearly identical.

## Abbreviations

CNV: copy-number variant; PEM: paired-end mapping; *PEMer*: *paired-end mapper*; SNP: single nucleotide polymorphism; SV: genomic structural variant.

## Authors' contributions

JK and MG conceived of the study, and participated in its design and coordination. JK and AA performed the simulations. JK, AA, XM, NC, PC, MS, and MG helped setup the *PEMer *pipeline. JK, AA, XM, NC, PC, ZZ, MS, and MG analyzed the processed paired-end sequence read data. JK, AA, XM, and MG wrote the manuscript. All authors read and approved the final manuscript.

## Additional data files

The following additional data are available with the online version of this paper: Additional data file 1 includes supplementary methods, Figures, and Tables.

## Supplementary Material

Additional data file 1 Supplementary methods, Figures, and Tables. Click here for file
