# Supplementary material for: PEMer: a computational framework with simulation-based error models for inferring genomic structural variants from massive paired-end sequencing data
Source: Genome Biol. 2009 Feb 23;10(2):R23. doi: 10.1186/gb-2009-10-2-r23 (PMC2688268; doi:10.1186/gb-2009-10-2-r23)
Supplement: Additional data file 1 — Supplementary methods, Figures, and Tables. [file gb-2009-10-2-r23-S1.doc]

Supplementary Methods, Notes, Figures, and Tables for the Manuscript:

PEMer: a computational framework with simulation-based error models for inferring genomic structural variants from massive paired-end sequencing data


Jan O Korbel1,2,3,*, Alexej Abyzov3, Xinmeng Jasmine Mu4, Nicholas Carriero5, Philip Cayting3, Zhengdong Zhang3, Michael Snyder3,4, Mark B Gerstein3,4,5,6,*

1Gene Expression Unit, European Molecular Biology Laboratory (EMBL), Meyerhofstr. 1, Heidelberg, 69117, Germany; 2European Bioinformatics Institute (EMBL-EBI), Wellcome Trust Genome Campus, Hinxton, Cambridgeshire, CB10 1SA, UK; 3Molecular Biophysics and Biochemistry Department; 4Department of Molecular, Cellular, and Developmental Biology, 5Department of Computer Science, 6Program in Computational Biology and Bioinformatics, Yale University, New Haven, CT 06520, USA.

*Correspondence may be addressed to 
J.O.K. (korbel@embl.de) or M.B.G. (mark.gerstein@yale.edu)

Email-addresses of all authors are given below in the same order as in the author’s list:
korbel@embl.de, abyzov@gersteinlab.org, xinmeng.mu@yale.edu, nicholas.carriero@yale.edu, philip.cayting@yale.edu, zhengdong.zhang@yale.edu, michael.snyder@yale.edu, mark.gerstein@yale.edu


The optimal paired-end placement algorithm. When PEM data generated with the 454/Roche platform are processed, PEMer uses the optimal paired-end placement algorithm for combining ends initially mapped to a reference genome, and assessing whether the paired-ends can be reliably assigned to a unique genomic locus. Therefore, PEMer uses an adapted version of the placement algorithm described in ref. [29]. In particular, for each end processed in the read-alignment step, PEMer by default discards all but the 30 best-scoring hits to the genome, and subsequently assesses whether an optimal placement of combined ends is feasible by calculating the placement score: First, PEMer awards end-matches with highest sequence identity when aligned to the reference genome a score (i.e., ‘+1’). Second, PEMer scores the longest sequence alignments (+1). Third, PEMer rewards scores to each end, respectively, if it maps to the genome with allelic levels of sequence identity, i.e., at ≥99.5% (+1). Fourth, to avoid SV misassignment because of closely related (e.g., paralogous) sequences in the genome, PEMer assigns a penalty to paired-end placements in case there exists besides a putative outlier placement ‘A‘ another placement ‘B’ with nearly identical alignment quality that represents a non-outlier (–2). For this purpose, PEMer by default assumes match ‘A’ is not nearly identical with match ‘B’, if  ”length of (match) ‘A’” – 2 ≥”length of (match) ‘B’” and ”sequence identity ‘A’” – 2% ≥ “sequence identity ‘B’”. Fifth, PEMer penalizes paired-end placements in which ends match onto different strands (–2), in order to avoid calling inversions in case there is a nearly as good match in the genome that would be inconsistent with an inversion. Only paired-ends revealing a single (unique) best overall placement score are reported and all other paired-ends discarded by PEMer.
In conclusion, the optimum paired-end placement step discards outlier placements in case they are not robust, and potential misalignments owing to base-calling errors, or SNPs, are thus to some extent compensated for. At later stages (i.e. at the outlier-clustering step), additional filtering procedures may optionally be employed to receive higher confidence SV calls: i.e., PEMer may require that with each cluster of paired-ends considered, at least for one paired-end both ends must align to the reference genome at a sequence identity ≥97%. Furthermore, due to the rare event of false-negative genomic alignments when using Megablast or BLAT, PEMer may optionally require that within each cluster, for at least one paired-end each end must yield a best-scoring sequence alignment genome-wide when using the alternative indexing algorithm (i.e., using Blat in case Megablast was used initially in the read-alignment, and vice versa). Furthermore, PEMer may optionally require support for SV-calls from different PEM libraries or from different next-generation sequencing platforms.
	When processing Solexa/Illumina or SOLiD/ABI paired-end data, PEMer, rather than implementing the optimal paired-end placement step described above, considers paired-ends as optimally placed if they are confidently aligned onto the human genome as assessed using MAQ mapping qualities (see Methods).
 
Effect of chimeric paired-end inserts. One potential issue with PEM protocols requiring circular DNA intermediates, such as the one from 454/Roche, is the generation of chimeric paired-ends that may potentially lead to false positive SV assignments. For example, it was recently reported that a small fraction (i.e. less than 2%) of the paired-ends generated by the common 454/Roche protocol are chimeric [21]. To consider this potential source of error, we included in PEMer the functionality to monitor the false positive rate due to chimera formation by simulation. Specifically, we conservatively assumed that for an extremely high span-coverage (λ=25x), about 2% of paired-ends are chimeric, i.e. in 2% of all paired-ends the sequenced ends stem from different original inserts and thus represent a potential positive source of false positives. An upper bound estimate for the chimera-specific false-positive rate was obtained after considering the fact that the vast majority of ends stemming from chimera map to distinct chromosomes and are thus initially discarded by PEMer. In particular, the fraction of chimera mapping to the same chromosome is expected to be highest for chromosome 2, the chromosome containing the largest amount of sequenced DNA, i.e. 237 Mb (approximately 8% of all human DNA). We calculated an upper-bound estimate using chromosome 2, by applying the condition that a maximum of =0.016% paired-ends are both chimeric and map to the same chromosome. We did not observe any false-positives in our simulations using these criteria. Thus, we conclude that at least when carrying out PEM experiments with the 454/Roche platform, chimera-formation has a negligible effect on the false positive rate. 

Considerations concerning span-coverage and paired-end cluster size. We use the Poisson approximation [45] to determine the likelihood that a SV in a sample genome is covered by a cluster of N paired-ends given a certain span-coverage. The span-coverage λ is n * L / G, with n being the number of paired-ends mapped onto to the genome, L being the median paired-end span, and G being the size of the genome analyzed (note that G usually refers to the euchromatic regions of the diploid human genome, i.e. approximately 6 Gb; in our simulations G refers to the euchromatic regions of the diploid chromosome 2, i.e. 237 Mb). With k being the number of observations of a particular SV, the probability P of covering a certain genomic element k times is:
						Equation [1].
Here we equate the probability of covering a genomic element with the probability of detecting a SV. In practice, a SV must be covered at least N times by a paired-end in order to be called by PEMer, with N being the cluster size. For N≥2, P can be calculated as follows. P(k<2; λ = P(0; λ + P(1; λ. As P(k ≥ 2; λ = 1– P(k<2; λ, we can write Equation [2] as:
P(k≥2; λ = 			Equation [2]. 
Thus, with λ = 5 and N = 2, P(k≥2; 5) = 1-(1+5) e-5 = 96%. Thus, at 5x span-coverage 96% of all SVs are expected to be detected when a cluster size N=2 is required. Conversely, in order to identify 95% of all heterozygous SVs in a diploid genome, at least 4.75x of the diploid genome will need to be effectively spanned by SVs. Obviously, Equations [1] and [2] can be easily extended to determine also the probability, depending on l for identifying SVs at cluster sizes that are larger then 2. Equation [3] exemplifies this for N=3:
P(k<3; λ = P(0; λ + P(1; λ + P(2;l), and P(k≥3; λ=1- P(0; λ - P(1; λ - P(2; λ, i.e.:
P(k≥3; λ = 				Equation [3].
Thus, for example, with λ = 5 and N = 3, P(k≥3; 5) = =88%.

Initial comparison of SV-calls based on PEMer with calls based on another computational approach. While this paper was in preparation, Lee and coworkers [39] published an approach that allows detecting SVs from paired-end sequence reads based on Sanger sequencing data, rather than based on next-generation sequencing data. Lee et al. applied their approach to detect SVs in the diploid genome of Dr. J. Craig Venter [19] relative to the human reference genome (hg18). We regard it likely that both PEMer and the approach by Lee et al., including concepts described in both studies, are going to be applied and concept-wise extended in the future. In this regard, a comparison of both approach is likely of interest to the community. However, at this point, only a rather preliminary comparison of both approaches is possible and results of the comparison should be regarded with caution, for several reasons: thus far, the approaches have been applied with data from different sequencing platforms generated using different paired-end protocols, and the genomic DNA both studies focused on were from different individuals. Nevertheless, to allow at least a preliminary comparison of both datasets, we have used the Galaxy-browser (http://main.g2.bx.psu.edu/) to intersect calls from both algorithms with SVs reported in the Database of Genomic Variants (DGV). In particular, we prepared a custom version of DGV by removing SV-calls that were detected when analyzing the genomes of NA15510, NA18505 (which were analyzed in this study), and Dr. J. Craig Venter (analyzed by Lee et al.). We then applied the dataset intersection tool from the Galaxy-browser with default parameters (i.e. using the options: “overlapping intervals” and “1 bp overlap”) to calculate the overlaps of deletion and inversion events with events reported in DGV. We did not compare insertion-calls as the detection ranges for insertions generated by both approaches show little overlap. Out of the 2988 deletions and inversions reported by Lee et al., 51% intersected with SVs in the DGV. Furthermore, when applying alternative scoring criteria to the Lee et al. data, by considering SV-calls only if they are supported by at least one uniquely mapping mate-pair [39], 45% of the remaining set of SVs from Lee et al. intersected with DGV-calls. In contrast, 74% out of 622 SVs (deletions and inversions) that PEMer identified in NA18505 intersect with SVs reported in DGV. Furthermore, 72% out of 353 SVs that PEMer identified in NA15510 intersect with SVs reported in DGV. Although these results are difficult to interpret for the reasons given above, they may indicate a comparably higher specificity of PEMer SV-calls compared to the Lee et al. calls; note that most SVs present in the genome are expected to represent common polymorphisms and thus they should be identifiable across studies and individuals [22]. On the other hand, it is possible that many of the seemingly novel SVs identified in Lee et al. represent common true positive SVs that were missed by previous SV mapping studies.


Figure S1. Diagram of the BreakDB schematic. Database tables and their relationship are illustrated.


Figure S2. Distribution of signal frequencies for homopolymers of different length in 454-Sequencing data. Signal distributions are given for the background (black curve), for single nucleotides (”homopolymer size” of 1), and for homolpolymers of sizes 2 to 9.


Table S1. Cutoffs C used for given cluster sizes N at span-coverages . 
Cluster size / Span-coverage	2	3	4	5	6	7	8	9	10	11	12
5x	3.4-3.5	3.0-3.1	2.7-3.0	2.4-2.7	2.2-2.4	2.2-2.3	2.0-2.1				
8x	3.5-3.6	3.1-3.2	2.8-3.0	2.7-3.0	2.5-2.6	2.4-2.5	2.3-2.4	2.1-2.3	2.0-2.2		
10x	3.5-3.9	3.1-3.2	2.8-3.0	2.7-2.8	2.6-2.7	2.5-2.6	2.4-2.5	2.2-2.3	2.1-2.2	2.0-2.1	
15x	N/A	3.2-3.8	3.2-3.4	3.0-3.1	2.6-2.8	2.5-2.6	2.4-2.5	2.3-2.4	2.3-2.4	2.3-2.4	2.2-2.3
20x	N/A	N/A	3.2-3.4	3.0-3.1	2.7-2.8	2.6-2.7	2.6-2.7	2.4-2.5	2.4-2.5	2.4-2.5	2.3-2.4
25x	N/A	N/A	N/A	3.2-3.4	2.8-3.1	2.8-2.9	2.7-2.8	2.5-2.6	2.5-2.6	2.4-2.5	2.3-2.4


The given ranges indicate cutoffs for which the false positive rate was ~5% – ~0%, according to our simulations. N/A, no cutoffs were reported as the number of observed false-positive calls was extensive. Paired-ends with a span deviating from the median insert size by less than 2 standard deviations were not considered in this simulation.
 


Table S2. E-values for predicted SVs. 
C / N  - deletions	2 overlaps	3 overlaps	4 overlaps	5 overlaps	6 overlaps
2.0	52248	12064	3061	824	231
2.5	4500	327	26	2.2	0.2
3.0	246	4.5	0.1	1.9e-3	4.3e-5
3.5	8.4	3.1e-2	1.2e-4	5.2e-7	2.3e-9
C / N  - insertions	2 overlaps	3 overlaps	4 overlaps	5 overlaps	6 overlaps
2.0	16315	1492	161	19	2
2.5	1051	23	0.58	1.6e-2	4.9e-4
3.0	43	0.2	8.3e-4	4.4e-6	2.5e-8
3.5	1.1	6.7e-4	4.8e-7	3.8e-10	3.2e-13
inversions	2 overlaps	3 overlaps	4 overlaps	5 overlaps	6 overlaps
	~0*	~0*	~0*	~0*	~0*


E-values depend on the number of paired-ends in a cluster and the length of the shortest paired-end span. Values are given for a span- coverage of =5x (2500 bp insert size; simulations according to the 454/Roche protocol). E-values for deletions and insertions are according to analytical equations presented in the Methods section. *E-values for inversions are based on simulations carried out at =5x; these simulations did not reveal any false positive inversions. Note however that in additional simulations carried out at very high coverage (i.e., at ≥15x) we did observe some false-positive inversions. Note in addition that the relative impact of base-calling errors on inversions is higher than the relative impact of base-calling errors on insertions or deletions, as false positive inversions are usually generated by misaligned reads whereas false positive insertions and deletions are mostly due to (correctly aligned) clustered random outliers.


 Table S3. Efficiency of PEMer for reconstructing heterozygous deletions using the 454/Roche platform with different span-coverages. 
SV-reconstruction with a reasonable rate of false positives of ~5%.
SV size / 	5x	8x	10x	15x	20x	25x
1000	3	5	6	4	4	4
2000	11	15	21	33	43	59
3000	49	67	80	91	96	97
4000	80	90	93	95	95	95
5000	91	97	98	97	97	97
6000	92	99	99	98	98	98
10000	88	97	97	98	98	98
Total	414	470	494	516	531	548
False positives (chromosome 2)	5	8	7	7	6	7

SV-reconstruction with near-zero false positives.
SV size / 	5x	8x	10x	15x	20x	25x
1000	3	5	6	4	4	3
2000	7	12	14	23	39	50
3000	38	58	73	90	94	97
4000	77	90	93	92	95	95
5000	89	96	97	97	97	97
6000	92	99	99	99	99	99
10000	88	97	97	98	98	98
Total	394	457	479	503	526	539
False positives (chromosome 2)	0	0	1	2	0	0


Following simulations, SV-reconstruction was carried out with cutoffs C given in Table S1 using two alternative parameter settings. The median insert size was 2500 bp.


Table S4. Reconstruction efficiencies for simulated heterozygous inversions using the 454/Roche PEM protocol with a median insert size of 2.5 kb

Inversion size	Reconstructed out of 100
1000	83
2000	96
3000	98
4000	97
5000	97
6000	97
10000	97


The span-coverage was =5x. No false positive inversions were obtained on chromosome 2 at this span-coverage, and neither at =10x (near-zero false positive rate). Note that the relatively high rate of inversions reconstructed (e.g. compare with Table 1) can be explained by the fact that both breakpoints occurring in a single inversion event are detectable by discordant paired-ends (thus, the specific effective coverage at inversion breakpoints can approximately be equated with 2λ). Note that even if only a single breakpoint is spanned by paired-ends, both breakpoints can be deduced within the range of the insert size – i.e. within ~2.5 kb in the case of the 454/Roche paired-end platform.


Table S5. Reconstruction efficiencies for simulated heterozygous insertions using the 454/Roche paired-end platform with a median insert size of 2.5 kb
Insertion size	Out of 100 reconstructed
250	0
500	1
750	2
1000	1
1250	8
1500	3
1750	3
2000	1
2250	1
2500	0
2750	0
3000	0


The span-coverage was =5x. Cutoffs (multi-cutoff strategy) were adjusted to achieve a false positive rate ~0%.

Table S6. Effect of insert size: simulated reconstruction efficiency for heterozygous insertions and deletions at 5x coverage using the 454/Roche paired-end platform with 10 kb inserts

SV size	Reconstruction efficiency for deletions	Reconstruction efficiency for insertions
1000	13	8
2000	47	42
3000	77	72
4000	87	69
5000	94	61
6000	89	55
7000	(not tested)	37
8000	(not tested)	23
9000	(not tested)	4
10000	87	1
Total	494	372
False positives (chromosome 2)	4	4


We simulated PEM with 454/Roche platform data using a 10 kb insert size and a standard deviation identical to the standard deviation observed in data from the “1000 Genomes Project” [47] for the 2.5 kb protocol. We applied the following cutoffs for clusters of N=2 paired-ends: 3.3; N=3: 2.6; N=4: 2.5; N=5: 2.4; N=6: 2.3; N=7: 2.0 (false positive rate of ~5%). Applying paired-ends with 10kb, rather than 2.5 kb, insert size, led to an increased range at which insertions can be reconstructed and further to an improvement in insertion reconstruction efficiency (compare with Table S5). In addition, deletions were reconstructed at high efficiency (compare with Table S3). Note further that sequencing with long insert sizes is relatively economic, as already relatively small sets of paired-ends lead to comparably high values of . 

Table S7. PEMer reconstruction efficiency for homozygous deletions. 
SV size	Single cutoff	Multi-cutoff	Simplified  multi-cutoff
1000	5	7	5
2000	28	59	16
3000	82	90	82
4000	95	94	94
5000	97	98	97
6000	98	99	99
10000	97	97	97
Total	502	544	490
False positives	31(31)	5(4)	2(1)


A diploid chromosome 2 was analyzed at 5x span-coverage (2500 kb median PEM insert size; simulations according to the 454/Roche based PEM protocol).


Table S8. Optimal cutoffs C for the Solexa/Illumina PEM protocol
Cluster size N	2	3	4	5
Cutoff C	3.5	2.9	2.6	2.3


Simulations were performed with realistic parameters with regard to primary data generated by the Solexa/Illumina sequencing platform, i.e. assuming a mean paired-end span of 250 bp, a normal distribution of spans, and a standard deviation of ~25 bp. The effective span-coverage was =5x – in particular,  was assessed following optimal paired-end placement (using MAQ) and thus represents the effective span-coverage. Cutoffs were determined assuming a ~5% false positive rate (see also Table S9).


Table S9. Reconstruction efficiency for simulated paired-ends based on the Solexa/Illumina platform. 
Deletion size	Single cutoff [Reconstruction efficiency (%)]	Simplified multi-cutoff [Reconstruction efficiency (%)]
100	56	68
200	81	81
300	80	80
400	85	85
500	81	81
5000	91	91
10000	88	88
False positives (chromosome 2)	7	20


PEMer was used for scoring simulated Solexa/Illumina PEM data with parameters and cutoffs described in Table S8. SVs were reconstructed at an efficiency comparable to the 454/Roche platform (compare with Table 1); note, however that at this point we did not specifically focus on SVs embedded within repeats (i.e. such formed by NAHR) in which the 454/Roche platform is expected to perform at comparably higher efficiency due to the better alignment specificity of longer reads. Mapping was carried out with MAQ; in this simulation all base scores were set to 70. Cutoffs were set to achieve a false-positive rate of ~5%. In this regard, note that according to a recent study (i.e., ref. [19]; see tables “HuRef.InternalHuRef-NCBI.gff” and “HuRef.homozygous_indels.inversion.gff”), ~6000 SVs of at least 100 bp in size (the size-range of Solexa/Illumina PEM) are expected per diploid genome; we thus applied 6000 as scaling factor to estimate the genome-wide false positive rate as described in the Methods section.


Table S10. Comparison of BLAT and Megablast on 454/Roche PEM data. 
 
Event length	Megablast	BLAT	BLAT(*)
1000	3	7	6
2000	27	21	22
3000	66	58	71
4000	87	86	88
5000	95	92	94
6000	94	92	92
10000	93	88	92
Total	465	444	465
False positives	3	12	8
Effective  span-coverage	5.4x	4.6x	5.2x


SV reconstruction was simulated using a monoploid chromosome 2 with 7x effective span-coverage, cutoffs C=3.3 for N=2; C=2.8 for N=3; C=2.4 for N=4; C=2.0 for N=5. Megablast (run with options: -p 80 -s 11 -W 11 -M 1000000) was more sensitive than BLAT (run with default options), at the cost of an over 3-fold increased run time. BLAT run with an alternative option (–minMatch=1 (*)) was 20% faster than Megablast, and was slightly less sensitive. Note that the slightly higher mapping-rate of BLAT compared to Megablast and the slightly increased sensitivity of Megablast compared to Blat were observed for several parameter sets both algorithms were tested with. Thus, both methods have their advantages and disadvantages and may be used depending on the required application. The table indicates how many heterozygous deletions at different size ranges – out of 100 simulated ones, respectively, were reconstructed when using BLAT or Megablast as indexing algorithms.


Table S11. PEMer timing data. 
General statistics	Total
Total number of 454 sequencing runs	122
Total number of sequenced paired-ends	73,719,731
Total number of jobs (bundles of 400 reads)	184,429
Bundles set with perfect 454-linker match (set 1)	56,899
Bundles set lacking perfect 454-linker match (set 2)	127,530
	
Timing data for set 1	CPU hrs
Linker mapping	- *
Initial alignment	24,289
Rapid indexing using the Megablast algorithm	14,975
Realignment using the Smith-Waterman algorithm	9,314
Optimal placement	112
Outlier identification	9
Total set 1	24,411
	
Timing data for set 2	CPU hrs
Linker mapping	944
Initial alignment	2,353
Rapid indexing using the Megablast algorithm	2,027
Realignment using the Smith-Waterman algorithm	326
Optimal placement	21
Outlier identification	4
Total set 1	3,322
	
Sum	CPU hrs
Total sets 1 and 2	27,732


Timing data were recorded while mapping SVs in a sample sequenced by the “1000 Genomes Project” [47] with the 454/Roche platform. 74 M paired-ends with a median fragment size of 2.5 kb were sequenced, 27 M of which were optimally placed. Paired-ends were divided into two sets: set 1, i.e. 23 M fragments with identical match to the 44 bp linker sequence, and set 2, i.e. 51 M fragments lacking an identical linker sequence (some of these had a recognizable linker matching with sequence identity <100%). Note that as parameter estimation, outlier-clustering, and cluster-merging do not represent the time-limiting steps of PEMer, they were omitted in this table. For set 1, linkers had been mapped at Baylor College of Medicine, Houston, Texas, prior to our analysis. We estimated based on the number of placed paired-ends in set 2 (i.e. ~2 M) that 5×3,32216 k CPU hrs are required for processing 10 M paired-ends.

Table S12. List of additional SVs identified when rescoring published datasets 

ID	Sample	Genomic coordinates	Intersection with DGV	Formation mechanisms	Inferred type(*)
SV_1	NA15510	Chr15:66212941-66216340	Yes	SVA	Insertion
SV_2	NA15510	chrX:154570467-154574981	Yes	Unknown	Deletion
SV_3	NA18505	Chr15:18280799-18286733	Yes	Satellite	Expansion
SV_4	NA18505	chrX:61604319-61608491	-	Satellite	Expansion
SV_5	NA18505	Chr21:9723652-9726629	Yes	Satellite	Expansion
SV_6	NA18505	Chr5:2021062-2024785	Yes	LINE	Insertion
SV_7	NA18505	Chr9:109575465-109581064	Yes	SVA	Insertion
SV_8	NA18505	Chr14:22165514-22169443	Yes	unknown	Deletion
SV_9	NA18505	Chr14:39165768-39170010	Yes	LINE	Insertion
SV_10	NA18505	Chr17:20551592-20555787	Yes	unknown	Deletion
SV_11	NA18505	Chr18:15374221-15379039	-	Satellite	Expansion
SV_12	NA18505	Chr18:61917635-61921121	Yes	unknown	Deletion
SV_13	NA18505	Chr2:132683496-132684419	Yes	Satellite	Expansion
SV_14	NA18505	Chr20:32705386-32709166	Yes	unknown	Deletion
SV_15	NA18505	Chr5:174061936-174066427	Yes	NAHR(**)	Deletion
SV_16	NA18505	Chr9:122295033-122300424	Yes	SVA	Insertion
SV_17	NA18505	chrX:5064343-5067814	Yes	NHEJ or FoSTes (**)	Deletion
SV_18	NA18505	Chr19:32425875-32426168	-	Satellite	Expansion


SVs were called with the simplified multi-cutoff strategy. For all SVs identified in addition to ref. [21], independent evidence was obtained by mining DGV [33] or by deducing plausible mutational mechanisms [16, 21, 22] likely involved in SV-formation through analyzing the genomic coordinates of SVs with the UCSC browser (http://genome.ucsc.edu/). Several SVs were consistent with human-specific SVA or LINE/L1 retrotransposon insertions (see e.g. [34]), or with satellite DNA expansions. (*) Occurrences of deletions, insertions, and satellite expansions relative to the ancestral primate genome were inferred by comparing the hg18 assembly with the genome assemblies of chimpanzee, orang-utan, and macaque (using the UCSC browser). (**) In two cases, inferred SVs were consistent with previously described SVs for which breakpoint sequences were already available [17]; in both cases, breakpoint junctions were analyzed using BLASTN [46] to infer likely mutational mechanisms as described in ref. [21]. ‘Unknown’ indicates that no breakpoint junctions could be deduced from published SV data and we found no compelling evidence for the involvement of retrotransposons or satellite repeats. The inferred mechanisms suggest that most predicted SVs are at high confidence (i.e. indicating a low false positive rate).
